# Effect of different pressure-targeted modes of ventilation on transpulmonary pressure and inspiratory effort

**DOI:** 10.1186/2197-425X-3-S1-A825

**Published:** 2015-10-01

**Authors:** N Rittayamai, F Beloncle, R Waheed, L Chen, M Rauseo, G Chen, EC Goligher, JCM Richard, L Brochard

**Affiliations:** Li Ka Shing Knowledge Institute and Critical Care Department, St. Michael's Hospital, Toronto, Canada; Interdepartmental Division of Critical Care Medicine, University of Toronto, Toronto, Canada; Division of Respiratory Diseases and Tuberculosis, Department of Medicine, Faculty of Medicine Siriraj Hospital, Bangkok, Thailand; Department of Intensive Care and Hyperbaric Medicine, Angers University Hospital, Angers, France; Department of Medicine, Division of Respirology, University Health Network, Toronto, Canada; Department of Physiology, University of Toronto, Toronto, Canada; Emergency Department, General Hospital of Annecy, Annecy, France

## Introduction

Spontaneous breathing during mechanical ventilation improves gas exchange and might prevent ventilator-induced diaphragm dysfunction. In pressure-targeted modes, transpulmonary pressure (P_L_) is the sum of pressure generated by the ventilator and muscular pressure. When inspiratory effort increases, P_L_ and tidal volume (V_T_) increase, potentially resulting in lung injury. This effect depends on the degree of inspiratory synchronization (i-sync); pressure-targeted modes can be classified into fully, partially, and non i-sync modes. A bench study [1] demonstrated that non-i-sync mode resulted in lower P_L_ and V_T_ than other modes, protecting the lungs from injury. We undertook to assess the effect of varying synchronization during pressure-targeted ventilation in critically ill patients.

## Objectives

To compare V_T_, P_L_, inspiratory effort (esophageal pressure-time product, PTP_eso_) and respiratory drive (airway occlusion pressure, P_0.1_) during three pressure-targeted modes with different degrees of i-sync.

## Methods

We conducted a randomized cross-over physiological study in spontaneously breathing ventilated patients. Twelve patients were enrolled (1 subsequently withdrew). Three pressure-targeted modes (Evita XL, Draeger, Germany) including fully (PC-CMV), partially (PC-SIMV), and non i-sync (APRV) modes were sequentially applied for 20 minutes in random order using the same driving pressure, PEEP and inspiratory time. Airway, esophageal, and gastric pressures, P_0.1_, and flow were recorded along with gas exchange and hemodynamics. P_L_ and PTP_eso_ were calculated.

## Results

**Figure 1:**
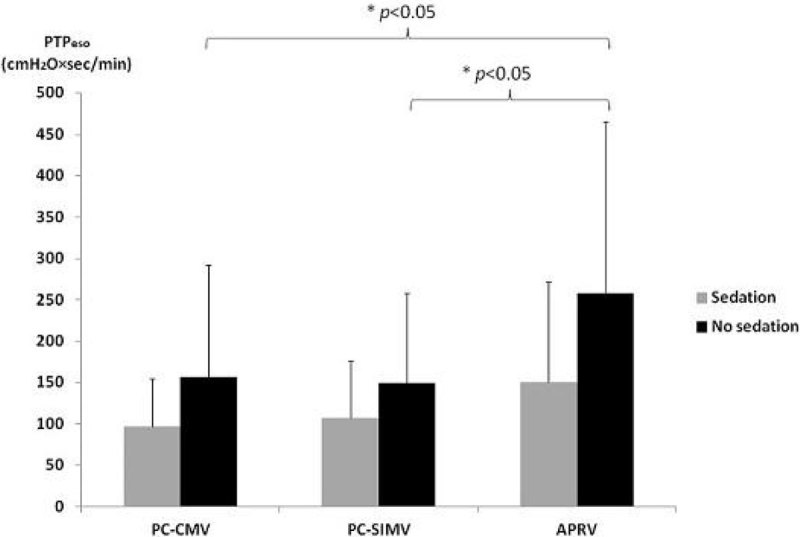
**Effect of intravenous sedation on PTPeso.**

11/12 patients successfully completed the study. V_T_ was significantly lower during non i-sync mode than fully i-sync mode (Table 1, *p* = 0.003) and V_T_ variability increased from 13 % to 35 % with decreasing inspiratory synchronization. Maximal P_L_ was significant lower in non-i-sync mode than in partially or fully i-sync modes (*p* = 0.008). There were no significant differences in gas exchange and hemodynamic parameters between modes. PTP_eso_ was significantly higher with non i-sync modes (Table 1, *p* = 0.047). This increase in PTP_eso_ was observed in the 6 patients who were not receiving intravenous sedation; no increase was observed in the 5 patients receiving continuous intravenous sedation (Figure 1).

**Table 1:** Table 1

	PC-CMV	PC-SIMV	APRV
Tidal volume per predicted body weight (mL/kg)	7.1 ± 1.0	6.5 ± 0.8	5.6 ± 1.2*
Maximal P_L_(cmH_2_O)	14.3 ± 4.5	14.0 ± 5.2	12.4 ± 4.8^*,#^
Minute ventilation (L/min)	10.4 ± 2.3	9.8 ± 2.0	9.9 ± 2.2
Breathing frequency (breaths/min)	21.6 ± 2.9	22.5 ± 3.9	26.9 ± 7.1*
PaO_2_/FiO_2_ ratio	221 ± 65	231 ± 57	227 ± 64
PaCO_2_ (mmHg)	48 ± 11	49 ± 12	50 ± 11
P_0.1_ (cmH_2_O)	2.6 ± 1.9	2.7 ± 1.7	3.9 ± 2.9
PTP_eso_(cmH_2_O*sec/min)	129.6 ± 107.1	130.2 ± 91.4	209.0 ± 174.9*

^*^ *p* < 0.05, PC-CMV vs APRV; ^#^ *p* < 0.05, PC-SIMV vs APRV

## Conclusions

Non synchronized pressure-targeted ventilation lowers V_T_ and P_L_ in comparison to fully and partially synchronized modes in spontaneously breathing ventilated patients, even with the same driving pressure. Appropriate sedation may be important to alleviate increased patient effort during such modes.

## Grant Acknowledgment

This study was supported by Dr. Brochard Laboratory Research Funding and by a grant from Siriraj Hospital in Bangkok, Thailand.
